# Utility and limitations of smartwatch electrocardiogram monitoring in a patient with idiopathic monomorphic ventricular tachycardia

**DOI:** 10.1016/j.hrcr.2025.03.024

**Published:** 2025-04-03

**Authors:** M. Kathryn McIntosh, Holly T. Philpott, Daniel V. Lancini, Ciorsti J. MacIntyre

**Affiliations:** 1 Dalhousie University, School of Medicine, Halifax, Nova Scotia, Canada; 2 Faculty of Medicine, University of Queensland, Queensland, Australia; 3 Heart Rhythm Service, Queen Elizabeth II Health Sciences Centre, Division of Cardiology, Halifax, Nova Scotia, Canada

**Keywords:** Ventricular tachycardia, Smartwatch, Polymorphic VT, Electrocardiogram, Monomorphic VT

**Key Teaching Points**

• Monomorphic ventricular tachycardia (VT) can appear as a more disorganized rhythm on smartwatch tracings, which may lead to misinterpretation and inappropriate management.
• Smartwatch ECGs should serve as an early detection tool, not a replacement for formal 12-lead ECG diagnostics, especially when evaluating serious arrhythmias.
• Patients should understand both the potential and limitations of smartwatch ECGs and seek medical evaluation for abnormal recordings or concerning symptoms.
• Although smartwatch ECG technology continues to improve, its role in clinical care should remain complementary to traditional diagnostic methods.

## Introduction

There is increasing utilization of smartwatch-based cardiac rhythm monitoring. Modern Apple Watches® (Apple Inc., Cupertino, CA) offer a single-lead electrocardiogram (ECG) recording, tailored to atrial fibrillation diagnosis.^1^, ^2^, ^3^ Their effectiveness in diagnosing ventricular arrhythmias remains underreported.^4^^,^^5^

The potential for monomorphic ventricular tachycardia (VT) to be misrepresented as a disorganized rhythm on Apple Watch tracings is of clinical significance given the divergent therapeutic and prognostic implications. Although smartwatch recordings have clinical utility in detecting abnormality, formal 12-lead documentation of arrhythmia should be sought.

## Case presentation

**Figure 1:**
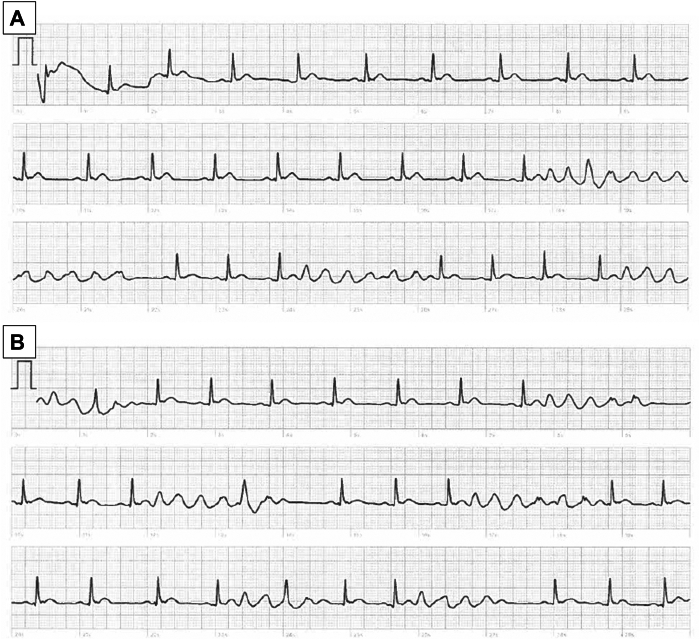
Apple Watch® single-lead ECG. The patient initiated recording on onset of palpitations by holding their finger to the digital crown of the watch for 30 seconds. This representative episode was recorded using the single-lead ECG function on their smartwatch (Apple Watch® Series 9, watchOS 10.0.2, Algorithm Version 2). The Apple Watch® recordings were from 23:10 and 23:11. The trace abnormality appears to represent polymorphic ventricular tachycardia (25 mm/s, 10 mm/mV, 512 Hz).

**Figure 2:**
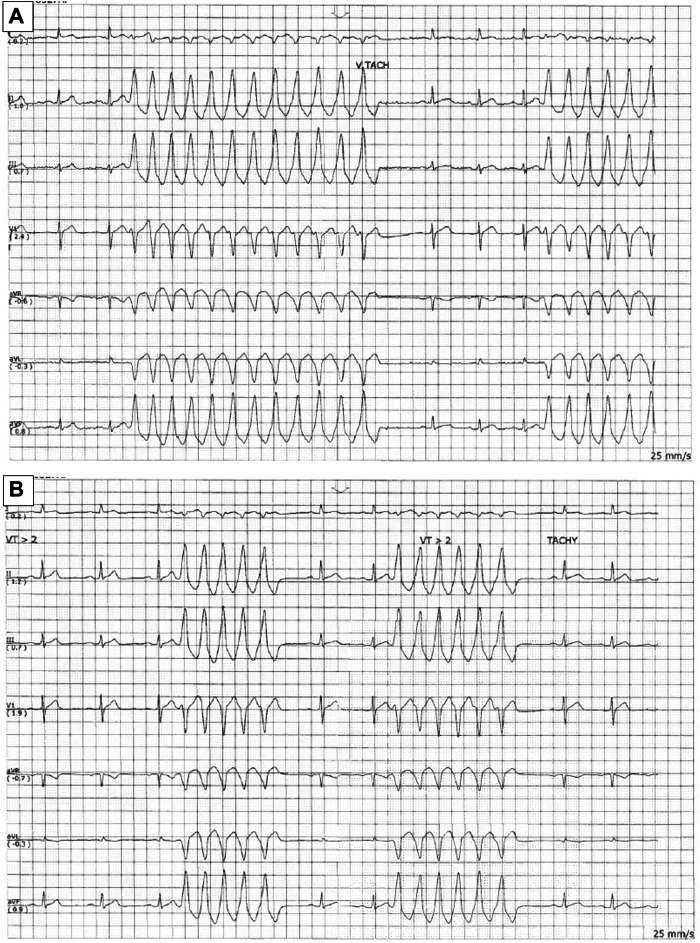
Standard in-hospital ECG tracings. A standard in-hospital ECG tracing obtained during admission. The tracings were taken at **A:** 23:15 and **B:** 23:21. The trace abnormality appears to represent monomorphic ventricular tachycardia. (25 mm/s, 10 mm/mV).

A 59-year-old man without prior cardiac history presented with recurrent palpitations and associated presyncope. Following manufacturer recommendations, he recorded these episodes using his Apple Watch® Series 9, by holding his right finger against the watch digital crown on his left wrist. These recordings displayed runs of disorganized, ventricular activity suggestive of polymorphic VT (Figure 1). However, further disorganized episodes on the smartwatch recorded during hospital admission, during the same episode of VT, displayed monomorphic outflow tract VT on 12-lead ECG (Figure 2).

## Discussion

Smartwatch ECGs, such as those on the Apple Watch, have shown promise in helping detect abnormal heart rhythms—especially for patients who might not experience symptoms during hospital monitoring. However, it is important to recognize both their potential and their limitations.

One key concern is that certain arrhythmias, such as monomorphic VT, can appear as more disorganized rhythms on smartwatch tracings. This misrepresentation matters because the treatment and prognosis for different arrhythmias vary significantly.

Although smartwatch ECGs can serve as a valuable early warning tool, they only capture a single lead and use different filter settings than the standard 12-lead ECG.^6^ This case highlights a diagnostically relevant discrepancy between smartwatch ECG recordings and standard 12-lead ECGs. This discrepancy raises important considerations regarding the signal processing and its differential effects across leads. The Apple Watch used in this case relies on proprietary algorithms, which presents a limitation in the analysis in this case. However, the knowledge of the potential for observed discrepancy alone is important. Future research into device processing settings will be an important step in understanding the interpretation of smartwatch-derived waveforms.

For these reasons, smartwatch ECGs should complement—not replace—traditional diagnostics. Whenever possible, a full 12-lead ECG should be obtained to confirm the findings. With ongoing research and education to raise awareness of these limitations, smartwatch technology can continue to support better heart health outcomes, provided it is used thoughtfully and alongside standard medical care.

## Disclosures

The authors have no conflicts of interest to disclose.
